# Model Complexity in Diffusion Modeling: Benefits of Making the Model More Parsimonious

**DOI:** 10.3389/fpsyg.2016.01324

**Published:** 2016-09-13

**Authors:** Veronika Lerche, Andreas Voss

**Affiliations:** Quantitative Research Methods, Institute of Psychology, Ruprecht-Karls-Universität HeidelbergHeidelberg, Germany

**Keywords:** diffusion model, *fast-dm*, parameter estimation, mathematical models, reaction time methods

## Abstract

The diffusion model (Ratcliff, 1978) takes into account the reaction time distributions of both correct and erroneous responses from binary decision tasks. This high degree of information usage allows the estimation of different parameters mapping cognitive components such as speed of information accumulation or decision bias. For three of the four main parameters (drift rate, starting point, and non-decision time) trial-to-trial variability is allowed. We investigated the influence of these variability parameters both drawing on simulation studies and on data from an empirical test-retest study using different optimization criteria and different trial numbers. Our results suggest that less complex models (fixing intertrial variabilities of the drift rate and the starting point at zero) can improve the estimation of the psychologically most interesting parameters (drift rate, threshold separation, starting point, and non-decision time).

The diffusion model (Ratcliff, 1978) is a popular mathematical model that recently attracted the attention of researchers of diverse fields of psychology (see Voss et al., 2013, for a recent review; see for example Brown and Heathcote, 2008, for another popular sequential sampling model). The model provides information about the cognitive processes underlying binary decision tasks. This becomes possible because the diffusion model parameters validly map specific latent cognitive processes (e.g., speed of information accumulation, decision bias). Despite the increased popularity of the diffusion model, there is a lack of research investigating how different model specifications influence the quality of the parameter estimation (see Lerche et al., 2016, for an exception). In particular, little to no information is available on the costs and benefits of model complexity. While the basic diffusion model (Ratcliff, 1978) comprises only four parameters, Ratcliff and Rouder (1998) and Ratcliff and Tuerlinckx (2002) suggested that it may be necessary to allow for intertrial variability of parameter values, because psychological processes (such as expectations or attention) will shift from trial to trial. This led to the inclusion of three so-called intertrial variability parameters.

Since then, these additional parameters have been estimated in almost all published diffusion model studies (e.g., Ratcliff et al., 2004b; Spaniol et al., 2008; Yap et al., 2012; Allen et al., 2014; van Ravenzwaaij et al., 2014), even if trial numbers were small to moderate (e.g., Metin et al., 2013). This might be problematic, because in this case the parameter estimation might become unstable.

The aim of the present article is to compare the performance of more parsimonious with more complex models. In doing so, we do not question the theoretic rationale of the intertrial variabilities. We are aware that in all applications there will be fluctuations in psychological processes. Nonetheless, we argue that sometimes the available data might not suffice to get reliable estimates for the full diffusion model. Thus, neglecting these fluctuations might lead to more accurate and stable results.

In the following sections, we first give a short introduction to the diffusion model. Then, we elaborate on necessary choices regarding estimation procedures and model specifications. Finally, we present data from a simulation study (Study 1) and from a test-retest study (Study 2).

## Parameters of the diffusion model

**Figure 1:**
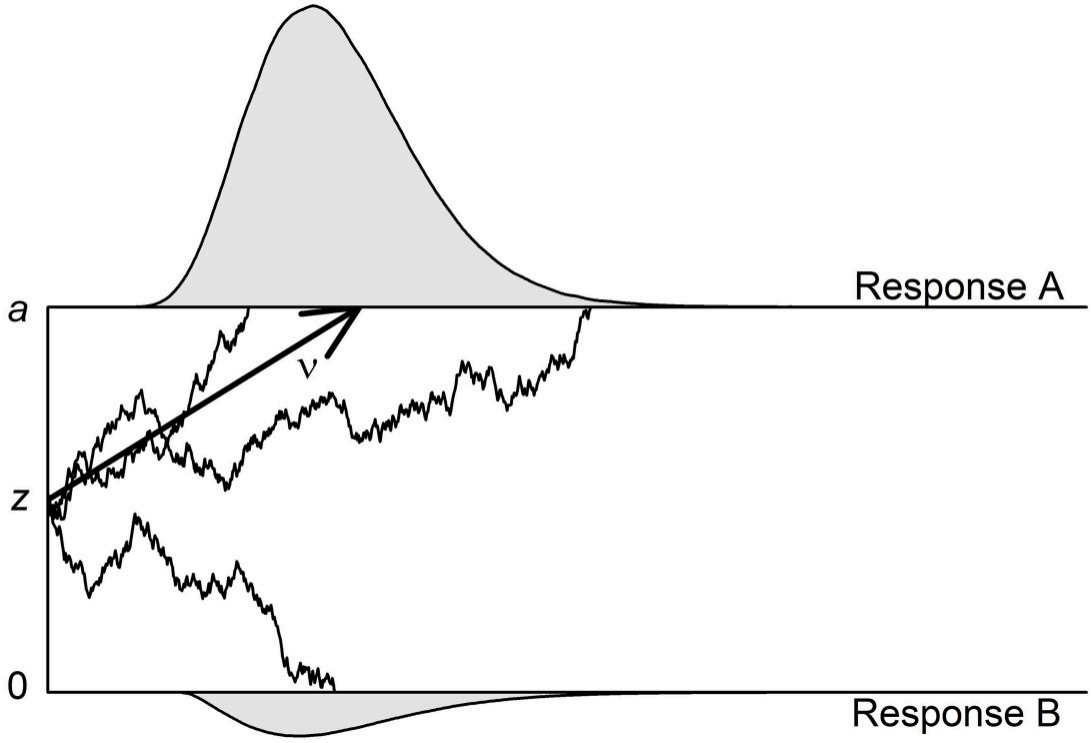
**Illustration of the diffusion model with three of its four main parameters**. The two thresholds that are associated with Response A (upper threshold; correct response in this illustration) and Response B (lower threshold; erroneous response) are separated by the distance *a*. The accumulation of information starts at starting point *z*, which is here centered between the thresholds. The mean drift rate (ν) is positive so that the upper threshold is reached more often than the lower threshold. In two of the three exemplary trials, the processes reach the upper threshold—resulting in one fast and one very slow correct response—and in one trial, the process reaches the lower threshold. The non-decisional component (*t*_0_) as well as the intertrial variabilities (*S*_*t*0_, *S*_ν_, and *S_Zr_*) are not depicted.

The diffusion model can be applied to binary decision tasks (e.g., lexical decision tasks [LDTs], or perceptual tasks such as color discrimination). One central supposition is that information is accumulated continuously and that this accumulation process ends as soon as one of two thresholds is reached. Each threshold is associated with one of the two responses of the binary task (or, alternatively, with correct vs. erroneous responses). Figure 1 shows an example of such a decision process.

The four parameters of the basic diffusion model are the (1) drift rate (ν), (2) threshold separation (*a*), (3) starting point (*z*), and (4) non-decision time (*t*_0_). The *drift rate* ν informs about the speed and direction of information accumulation. Positive (negative) drift rates indicate an average slope of information accumulation toward the upper (lower) threshold. The absolute value of the drift rate is a measure of the speed of information uptake with higher values indicating faster accumulation. The drift rate can be interpreted as a measure of subjective task difficulty: (absolute) drift rates will be higher for easier tasks. The diffusion model assumes that information uptake is a stochastic (i.e., noisy) process. Thus, the process does not necessarily end at the same time or at the same threshold, even if the same information is available.

The *threshold separation* (*a*) represents the chosen response criterion. Higher distances go along with longer information uptake and fewer erroneous responses. While in Figure 1 the process is assumed to start in the center between the two thresholds, it might also start at a position closer to the upper or lower threshold. If the *starting point z* (or, *z*_*r*_ = *z*/*a*) is located closer to one of two thresholds, less evidence needs to be accumulated before the participant decides for this option.

Finally, to the time taken by the decision process (illustrated in Figure 1) adds the *non-decision time t*_0_. It includes the duration of all processes that take place before (e.g., encoding of information) and after (e.g., motoric response execution) the decisional process. In most diffusion model studies one or more of these four parameters are in the focus of the research questions. Importantly, in several validation studies it was demonstrated that these parameters are sensitive to specific experimental manipulations, which supports the parameters' validity (e.g., Voss et al., 2004; Wagenmakers et al., 2008a; Arnold et al., 2015).

Ratcliff and Rouder (1998) suggest the inclusion of intertrial variabilities for two parameters, namely for the drift rate (*s*_ν_) and the starting point (*s*_*zr*_) (see also Laming, 1968, for an earlier account on intertrial variability). An important advantage of including these intertrial variability parameters in the model is that they provide an explanation for differences in speed of correct responses and errors. Specifically, if the *drift rate* varies from trial to trial, the model predicts *slower errors than correct responses*. Imagine trials with a drift rate that is higher than the average drift rate. In this case, all responses (including errors) are fast while the error rate is low. A drift rate that is lower than the average, on the other hand, results in a higher percentage of errors which are slow. Thus, the intertrial variability of the drift causes the majority of errors to be slow. A pattern of *faster errors than correct responses* can be explained by intertrial variability of the *starting point*. A starting point that is close to the lower (error) threshold increases the number of errors and decreases the decision time for those. If, on the other hand, the starting point is closer to the upper threshold (associated with correct responses), errors are slow but rare.

Later, a third variability parameter was included into the model: the intertrial variability of the non-decision time (*s*_*t*0_; Ratcliff and Tuerlinckx, 2002). A high intertrial variability of non-decision time accounts for a higher number of fast responses (i.e., the skew of the predicted RT distribution is reduced). Thereby, the model might also become less susceptible to the impact of fast contaminants. With the three intertrial variabilities, the diffusion model includes seven parameters (for a model with one further parameter, see Voss et al., 2010).

In most diffusion model studies intertrial variabilities are included not because they are important to answer a psychological research question, but rather to improve model fit and, possibly, to avoid a bias in the other parameters. In the present article, we test whether excluding the intertrial parameters derogates the estimation of the four main diffusion model parameters.

## Necessary choices in estimation procedures and model specifications

In the first decades after the introduction of the diffusion model in 1978, the parameter estimation was restricted to researchers with sound mathematical and programming skills. Now, several user-friendly software solutions exist that enable any researcher to apply a diffusion model to their data. Amongst these programs are *EZ* (Wagenmakers et al., 2007, 2008b; Grasman et al., 2009), *DMAT* (Vandekerckhove and Tuerlinckx, 2007, 2008), *fast-dm* (Voss and Voss, 2007, 2008; Voss et al., 2015), and *HDDM* (Wiecki et al., 2013). Even if these programs are easy to use, they require the users to make several choices in terms of the parameter estimation procedure (with the exception of *EZ* that works with closed-form equations and offers fewer degrees of freedom in model definition). One such choice regards the optimization criterion, another the complexity of the model (i.e., the number of estimated parameters).

### Optimization criterion

The diffusion model programs allow the choice between different optimization criteria. *Fast-dm-30* (Voss et al., 2015), for example, allows the choice between Kolmogorov-Smirnov (KS), a chi-square (CS) and a maximum likelihood (ML) based criterion. These criteria differ in the degree of usage of information with CS taking account of the least amount of information (RTs are grouped into bins) and ML using data from each single trial. On a continuum of information usage, with CS at the one end and ML at the other, KS can be positioned somewhere in between (see Voss et al., 2015, for a more detailed comparison of these three criteria). Related to information usage is the performance in parameter recovery. As a row of simulation studies by Lerche et al. (2016) shows, ML performs best, followed by KS and CS. The high efficiency of ML, however, comes with a cost: in the presence of fast contaminants (i.e., data not resulting from a diffusion process with the RTs situated at the lower tail of the distribution), the estimates obtained with ML are often severely biased. KS, on the other hand, turned out to be the least influenced by these contaminants.

### Model complexity

Most diffusion model programs allow an estimation of all seven parameters of the diffusion model. Furthermore, they also offer the possibility of fixing one or more of the parameters to a constant value, thereby specifying less complex models. As already mentioned, the intertrial variabilities are usually estimated not due to the theoretical interest in these parameters (see Ratcliff, 2008; Starns and Ratcliff, 2012, for an exception), but to avoid a biased estimation of the basic diffusion model parameters.

However, several simulation studies show that these parameters (especially, the variability of drift rate and starting point) are estimated less accurately than the other parameters (e.g., Vandekerckhove and Tuerlinckx, 2007; van Ravenzwaaij and Oberauer, 2009; Lerche et al., 2016). This raises the question of whether the inclusion of intertrial variability parameters really improves the estimation of the other parameters. Based on such findings, in some recent studies the intertrial variabilities have been deliberately fixed. For example, Germar et al. (2014) fixed all three intertrial variabilities at zero (see also Ratcliff and Childers, 2015). Note that also in earlier work the intertrial variabilities have sometimes been fixed at zero, because the application of the *EZ* method does not allow to include these parameters (e.g., Schmiedek et al., 2007; Wagenmakers et al., 2007, 2008b; Grasman et al., 2009; van Ravenzwaaij et al., 2012; Dutilh et al., 2013).

Whereas Ratcliff and Rouder (1998) and Ratcliff and Tuerlinckx (2002), who argued for the inclusion of intertrial variabilities, typically used very high trial numbers (at least 1000 trials per participant), more recently the model has also been applied to data sets with significantly smaller trial numbers (e.g., with only 100, see Metin et al., 2013). This raises the question of whether small data sets provide enough information to estimate the full (seven-parameter) model. Lerche et al. (2016) systematically investigated the number of trials that allow for a precise estimation of the diffusion model parameters. They simulated data sets both on the basis of a seven-parameter model (i.e., with the assumption of intertrial variabilities) and on the basis of more restricted models. For example, in a four-parameter model the three intertrial variabilities were fixed at zero both for the generation of data and for the reestimation of parameters. The comparison of these models revealed that—as expected—for more complex models higher trial numbers are required. Besides, as Lerche et al. (2016) show, the required number of trials also depends on the used optimization criterion. The authors found that the three optimization criteria KS, ML, and CS perform equally well for very high trial numbers. However, for small and moderate trial numbers, accuracy of estimates from CS based parameter search was inacceptable.

The findings by Lerche et al. (2016) raise the issue of whether less complex models (i.e., models with fixations) also perform better when the true (data generating) model is more complex (i.e., includes variabilities). A study by van Ravenzwaaij et al. (2016) speaks in favor of this hypothesis. The authors compared the performance of *EZ* (Wagenmakers et al., 2007) with the performance of a diffusion model estimation including all three intertrial variability parameters (using Quantile Maximum Proportion Estimation, see Heathcote et al., 2002). Interestingly, the power of between-group difference detection for both drift rate and threshold separation was higher for *EZ* than for the more complex model even if there were substantial intertrial variabilities in the data generating models. Thus, it seems that simpler models can outperform more complex models.

We further tackled this question in two studies, a simulation study (Study 1) and a test-retest study (Study 2). In Study 1, the performance of the estimation procedure is measured by deviations and correlations between the true and the recovered parameter values. In Study 2, the estimation performance is assessed by means of the correlations between the parameters of two different sessions.

## Study 1: simulation study

Study 1 is a simulation study in which we reanalyzed data sets of the seven-parameter model from Lerche et al. (2016).

### Method

Lerche et al. (2016) simulated data sets with different numbers of trials and reestimated parameters in order to deduce guidelines on requisite trial numbers. In Study 1, we reanalyzed a part of their data sets, namely the data sets that were created on the basis of the seven-parameter model (i.e., the model that includes intertrial variabilities and a bias in the starting point; see also Table 1). Here, we only briefly present their study design with a focus on the differences between the two studies. Please refer to Lerche et al. (2016) for more details on their simulation procedure.

**Table 1:** **Parameter ranges (Study 1) and means and standard deviations (Study 2) used for generation of parameter sets**.

**Parameter**	**Study 1: ranges**		**Study 2: *M(SD)***	
	**Minimum**	**Maximum**	**Lexical decision task**	**Recognition memory task**
a	0.5	2.0	1.42 (0.32)	1.60 (0.36)
ν	−4.0	4.0	−	−
ν_0_	–2.35 (1.0)^a^		–4.01 (1.13)	–3.07 (1.14)
ν_1_	2.00 (1.0)^a^		3.10 (1.11)	2.44 (1.20)
*t*_0_	0.2	0.5	0.48 (0.04)	0.61 (0.05)
Z_*r*_	0.3	0.7	0.53 (0.06)	0.55 (0.08)
*_Sν_*	0.0	1.0	1.34 (0.64)	1.41 (0.83)
S_*t*0_	0.0	0.2	0.15 (0.05)	0.17 (0.08)
S_*zr*_	0.0	0.5	0.37 (0.25)	0.15 (0.22)

*Parameter sets of Study 1/Study 2 were created on the basis of a uniform distribution/multivariate normal distribution, respectively. Fast-dm uses a diffusion coefficient of 1. For comparison with parameters used in studies with diffusion coefficient 0.1 multiply a, ν, z_r_, s_ν_, and s_zr_ by 0.1*.

a *The drift rates in the two-drift design were created on the basis of a multivariate normal distribution with the given means and standard deviations*.

The authors constructed data sets for two different experimental designs: a one-drift design and a two-drift design. Whereas the one-drift design simulates choices between two stimuli with the same absolute drift rate value, in the two-drift design the drift rate for one stimulus is larger than for the other stimulus (*d*_*z*_ = 0.35). Accordingly, in the one-drift design, only one drift rate was estimated. In the two-drift design, two drift rates (with opposite signs) were estimated simultaneously. One-thousand different parameter sets with random parameter values were used for each experimental design. For each parameter set seven data sets were created, using *construct-samples*^1^, with different trial numbers (24—48—100—200—500—1000—5000). Then, 4% of the simulated trials were randomly selected and substituted for by either fast or slow contaminants, resulting in three contamination conditions (no contaminants—fast contaminants—slow contaminants). More specifically, in the condition with fast contaminants, the responses of the contaminant trials were set by chance to 0 or 1 (simulating guesses) and the simulated RTs from these trials were substituted for by RTs situated at the lower edge of the original distribution (range: *t*_*min*_ − 100 ms to *t*_*min*_ + 100 ms, with *t*_*min*_ = *t*_0_ − *s*_*t*0_/2). In the condition with slow contaminants, only the response times were replaced, using values lying 1.5–5 interquartile ranges above the third quartile of the original RT distribution.

For each condition (stimulus design × trial number × contamination condition), Lerche et al. (2016) reestimated all seven parameters and compared them with their true values (in the remainder of this article termed “seven-parameter model”). In the present study, we additionally use more parsimonious models for parameter estimation. In particular, in the “five-parameter model”, two of the intertrial variabilities (*s*_ν_ and *s*_*zr*_) were fixed at zero (i.e., we assumed that these two parameters do not vary from trial to trial). We fixed these two intertrial variabilities, because several studies have shown that they are recovered poorly (e.g., van Ravenzwaaij and Oberauer, 2009). The intertrial variability of the non-decision time, on the other hand, is estimated better and could counteract the negative influence of fast contaminants. Thus, this parameter was kept in the model even if it is psychologically less interesting than the main diffusion model parameters (*a*, ν, *t*_0_, *z*_*r*_). Furthermore, we used a “four-parameter model” (i.e., the “basic” model) with additional fixation of the intertrial variability of the non-decision time (i.e., *s*_*t*0_ = 0). Note that these fixations are always false assumptions (“false fixations”), since the data generating model included all three intertrial variabilities. Finally, we estimated a “three-parameter model” in which we additionally fixed the starting point to the center between the two thresholds (i.e., *z*_*r*_ = 0.5). For the parameter estimation, we used *fast-dm-30* (Voss et al., 2015) and estimated the parameters with each of the three implemented optimization criteria (i.e., KS, ML, and CS).

Our evaluation criteria are similar to those by Lerche et al. (2016): We analyzed (1) correlations between the true and the reestimated parameter values, (2) biases (i.e., deviations between the true and the reestimated parameter values), and (3) estimation precision (i.e., squared deviations between the true and the reestimated parameter values). For criterion 1 and criterion 3 we additionally computed an average measure across parameters. Specifically, for criterion 1, we calculated the mean correlation over the four main diffusion model parameters using Fisher's Z-transformation^2^. The mean estimation precision was calculated on the basis of the formula stated below. Most importantly, differences between the estimated and the true parameter values were computed and weighted against the best possible accuracy that can be reached by each parameter. In contrast to Lerche et al. (2016), we computed the mean based on only the four basic diffusion model parameters (i.e., *a*, ν, *t*_0_, and *z*_*r*_)^2^,^3^.

$$mean\;estimation\;precision=\frac{1}{4}\cdot \sum_{\text{k}=1}^{4}{\left[\frac{{\text{estimated}}_{\text{k}}{-\text{true}}_{\text{k}}}{\text{best possible }{\text{accuracy}}_{\text{k}}}\right]}^{2}$$

If the interest of the researcher lies in relationships between the diffusion model parameters and external criteria, the correlation criterion is of most relevance. A disadvantage of correlation coefficients is that they can mask possible biases in parameter estimation (e.g., if a parameter is systematically over- or underestimated, still high correlation coefficients result). The bias criterion tackles such systematical deviations in parameter estimation. Finally, the estimation precision criterion is the strictest criterion, since it takes into account any inaccuracy in parameter estimation. This criterion is of relevance if the diffusion model parameters are to be used as diagnostic measures. Such a potential future use of diffusion model parameters requires very accurate parameter estimates.

### Results

**Figure 2:**
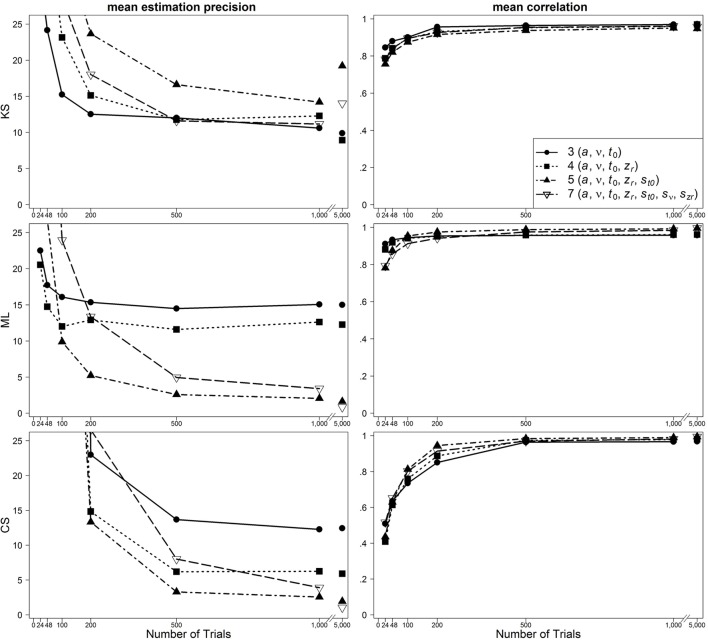
**Scatter plot of 95% quantiles of mean estimation precision (left column) and mean correlation between true and reestimated parameters (right column) for uncontaminated data sets in the one-drift design**. On the basis of data sets with at least 4% of trials at each threshold. Quantiles exceeding the mean estimation precision of 25 are not depicted.

**Figure 3:**
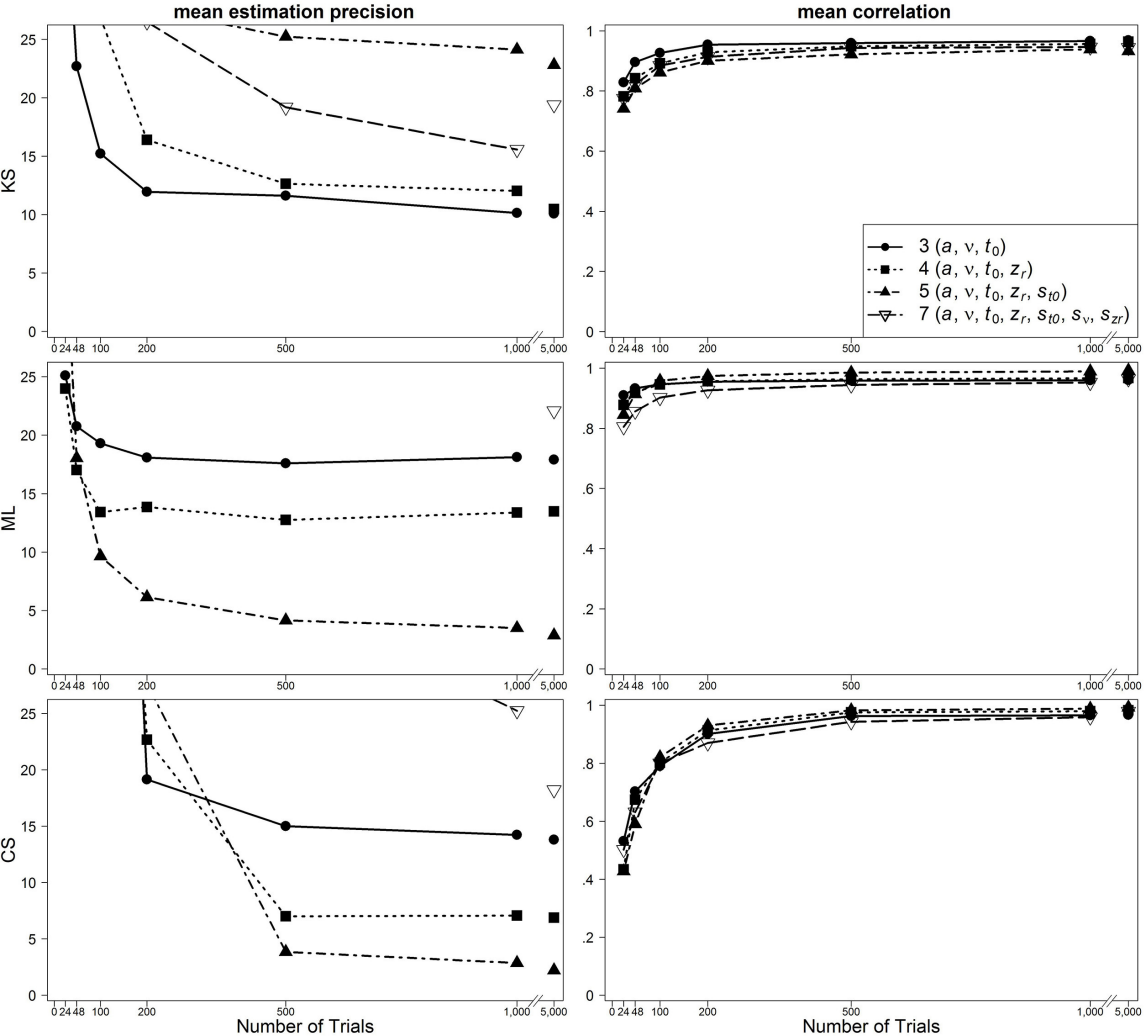
**Scatter plot of 95% quantiles of mean estimation precision (left column) and mean correlation between true and reestimated parameters (right column) for data sets with slow contaminants in the one-drift design**. On the basis of data sets with at least 4% of trials at each threshold. Quantiles exceeding the mean estimation precision of 25 are not depicted.

**Figure 4:**
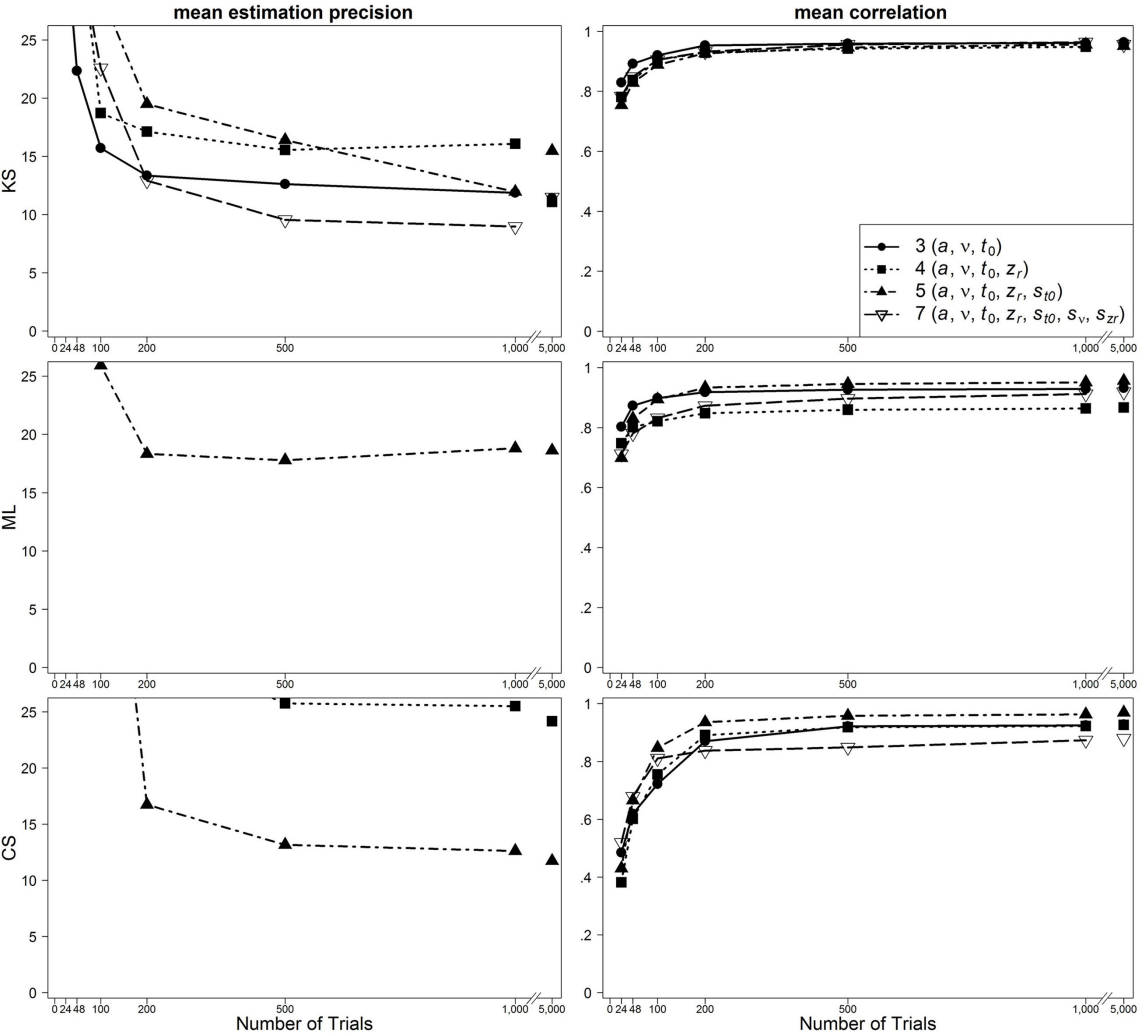
**Scatter plot of 95% quantiles of mean estimation precision (left column) and mean correlation between true and reestimated parameters (right column) for data sets with fast contaminants in the one-drift design**. On the basis of data sets with at least 4% of trials at each threshold. Quantiles exceeding the mean estimation precision of 25 are not depicted.

In Figure 2, results are presented for the one-drift design for uncontaminated data. Figures 3, 4 show results for the conditions of slow and fast contaminants, respectively. In the left column, the 95% quantiles of the mean estimation precision (criterion 3) are shown (thus, for most data sets, the mean estimation precision is smaller than the values from the figure). In the right column, mean correlation coefficients (criterion 1) are depicted. Results are presented as a function of number of trials, optimization criterion and model complexity^4^. Additionally, Table 2 (for the one-drift design) and Table 3 (for the two-drift design) sum up which model (model with 3, 4, 5, or 7 parameters) shows the best performance in terms of the correlations (first value), the mean bias across data sets (second value) and the 95% quantiles of estimation precision (third value) depending on the optimization criterion (KS/ML/CS), type of contamination (none/fast/slow) and number of trials. Note that in some conditions, several models manifest almost identical performance and that in these tables no information on the size of the differences between the models is given.

**Table 2:** **Model superiority for the one-drift design, depending on the type of contamination, the method, the parameter, and the number of trials**.

	**Method**	**Parameter**	**Number of trials**						
			**24**	**48**	**100**	**200**	**500**	**1000**	**5000**
None	KS	*a*	4/4/3	7/3/3	7/3/3	3/4/4	7/4/7	7/4/7	4/4/7
		ν	3/7/3	4/7/3	4/7/4	4/7/4	4/7/4	4/7/4	4/7/4
		*t_0_*	4/4/4	7/5/3	7/5/5	4/5/7	4/5/7	4/5/5	4/5/5
		*Z_r_*	7/4/4	4/4/4	4/4/4	7/4/4	7/4/7	7/4/7	4/4/4
	ML	*a*	3/3/3	3/3/3	5/5/5	5/5/5	5/7/5	5/7/5	7/7/7
		ν	4/4/3	4/5/4	4/4/4	5/4/5	5/4/5	5/4/5	7/7/7
		*t_0_*	3/4/4	3/5/5	5/5/5	5/5/5	5/5/5	5/5/5	5/7/7
		*Z_r_*	4/7/4	5/4/4	5/5/5	5/4/5	5/4/5	5/5/5	5/7/7
	CS	*a*	7/7/7	7/5/7	7/5/5	5/5/5	5/3/5	5/3/5	7/7/7
		ν	3/5/5	4/5/5	5/3/5	5/4/5	5/5/5	5/7/5	7/7/7
		*t_0_*	7/5/7	7/5/5	7/5/5	5/5/5	5/5/5	5/5/5	5/7/5
		*Z_r_*	7/7/7	7/4/7	7/4/7	5/4/5	5/4/7	5/4/5	5/4/7
Slow	KS	*a*	7/5/3	7/7/3	7/4/3	5/4/7	7/4/7	7/3/7	4/3/7
		ν	3/7/3	4/4/4	4/7/4	4/4/4	7/7/7	4/7/4	4/7/4
		*t_0_*	7/5/7	7/5/5	4/7/7	4/7/5	7/7/7	7/7/7	5/7/7
		*Z_r_*	7/4/4	5/4/7	5/4/7	7/4/7	7/4/7	7/4/7	7/4/7
	ML	*a*	5/5/5	5/5/5	5/5/5	5/5/5	5/5/5	5/5/5	5/5/5
		ν	4/4/4	4/4/4	4/4/5	5/4/5	5/4/5	5/4/5	5/4/5
		*t_0_*	7/7/7	5/7/7	5/7/7	5/7/7	5/7/5	5/7/5	5/7/5
		*Z_r_*	5/4/4	5/4/5	5/5/5	5/5/5	5/5/5	5/5/5	5/5/5
	CS	*a*	7/7/7	7/5/5	5/5/5	5/5/5	5/5/5	5/5/5	5/5/5
		ν	3/5/7	4/5/5	5/4/4	4/5/4	5/5/5	5/4/5	5/4/5
		*t_0_*	7/5/7	7/5/7	5/5/5	5/7/5	5/7/7	5/7/5	5/7/5
		*Z_r_*	7/4/7	7/4/7	5/4/7	5/5/5	5/7/5	5/5/5	5/7/5
Fast	KS	*a*	4/3/3	4/3/3	4/3/3	3/3/3	4/3/3	3/3/5	4/3/5
		ν	3/7/3	4/4/4	4/7/4	4/7/4	7/7/4	4/7/4	4/7/4
		*t_0_*	7/5/7	3/5/4	7/7/7	4/7/5	4/7/7	4/7/7	4/7/7
		*Z_r_*	4/4/4	4/4/4	4/4/4	4/4/4	4/4/4	4/4/4	4/4/4
	ML	*a*	3/5/3	5/5/5	5/5/5	5/5/5	5/5/5	5/5/5	5/5/5
		ν	4/7/3	4/7/4	4/7/4	5/4/4	5/4/4	5/4/4	5/4/4
		*t_0_*	4/5/4	4/5/5	5/7/5	5/7/5	5/7/5	5/7/5	5/7/5
		*Z_r_*	4/7/4	5/4/4	5/5/5	5/4/5	5/5/5	5/4/5	5/5/5
	CS	*a*	7/5/7	7/5/5	7/5/5	5/5/3	5/5/5	5/5/5	5/5/5
		ν	3/7/5	3/4/5	5/7/4	5/7/4	5/7/5	5/7/5	5/7/7
		*t_0_*	7/5/7	7/5/7	5/5/5	4/5/5	5/7/5	5/7/5	5/7/5
		*Z_r_*	4/4/4	4/4/4	4/4/4	4/4/4	5/5/5	5/4/5	5/7/5

*The first value is based on the correlation criterion, the second on the bias criterion, and the third on the estimation precision criterion. In the five-parameter model, the intertrial variabilities s_ν_ and s_zr_ are fixed at zero, in the four-parameter model additionally the intertrial variability s_t0_ is fixed at zero and in the three-parameter model also the starting point z_r_ is fixed (z_r_ = 0.5). For conditions with 95% quantiles of parameter estimation precision (weighted against the best possible accuracy) exceeding 25, values are depicted in gray. On the basis of data sets with at least 4% of trials at each threshold*.

**Table 3:** **Model Superiority for the two-drift design, depending on the type of contamination, the method, the parameter, and the number of trials**.

	**Method**	**Parameter**	**Number of trials**						
			**24**	**48**	**100**	**200**	**500**	**1000**	**5000**
None	KS	*a*	4/4/4	7/4/4	7/7/7	7/4/5	7/4/7	7/5/7	7/7/7
		ν	4/3/4	4/3/4	4/5/4	7/3/4	7/3/5	5/3/5	7/7/4
		*t_0_*	4/4/4	7/5/4	7/5/4	5/7/7	5/7/7	7/7/7	7/7/7
		*Z_r_*	7/4/4	7/4/4	7/4/4	7/7/7	7/7/7	7/7/7	7/4/7
	ML	*a*	7/5/5	7/5/5	5/5/5	5/5/5	5/5/5	5/7/7	7/7/7
		ν	4/4/4	4/4/4	4/5/4	5/5/5	5/5/5	5/7/5	7/7/7
		*t_0_*	4/5/4	5/5/5	5/5/5	5/5/5	5/7/5	5/7/7	7/7/7
		*Z_r_*	4/4/4	4/7/4	5/7/4	5/5/5	5/7/5	5/5/7	7/7/7
	CS	*a*	5/5/7	7/5/5	5/5/5	5/5/5	7/5/5	5/7/7	7/7/7
		ν	3/4/3	3/5/3	4/5/5	5/3/5	5/5/5	5/7/5	7/7/7
		*t_0_*	7/5/5	7/5/7	5/5/5	5/5/5	5/5/5	5/7/5	7/7/7
		*Z_r_*	4/4/4	7/7/4	7/7/4	7/7/5	7/7/7	7/4/7	7/5/7
Slow	KS	*a*	4/4/4	7/7/4	7/7/4	7/4/7	5/7/7	5/7/7	5/4/7
		ν	4/3/4	4/5/4	4/5/4	7/7/5	5/7/7	7/7/7	7/7/7
		*t_0_*	4/4/4	7/5/5	4/7/7	7/7/7	7/7/7	7/7/7	7/7/7
		*Z_r_*	7/4/4	7/4/4	7/4/7	7/4/7	7/4/7	7/4/7	7/4/7
	ML	*a*	4/5/5	5/5/5	7/5/5	5/5/5	5/5/5	5/5/7	5/5/7
		ν	3/4/3	3/5/3	5/5/5	5/7/5	5/7/5	5/7/5	5/7/7
		*t_0_*	7/5/7	7/7/7	5/7/7	5/7/7	5/7/7	5/7/7	5/7/7
		*Z_r_*	7/7/7	5/5/7	5/7/5	5/7/7	5/5/5	5/7/5	5/7/5
	CS	*a*	7/5/4	7/5/5	5/5/5	5/5/5	5/5/5	5/5/5	5/5/4
		ν	4/3/3	3/5/3	4/5/5	5/3/5	5/5/5	5/5/5	5/5/5
		*t_0_*	7/5/7	7/5/7	7/5/7	5/7/7	5/7/7	7/7/7	7/7/7
		*Z_r_*	4/7/4	7/7/4	7/5/7	5/5/7	7/5/7	7/7/7	7/7/7
Fast	KS	*a*	4/5/4	7/5/5	7/5/5	7/5/4	4/5/4	5/4/5	5/4/4
		ν	7/5/4	7/7/4	5/7/5	7/7/7	5/7/7	5/7/7	5/7/7
		*t_0_*	4/4/4	7/7/7	4/7/7	4/7/7	5/5/5	5/5/5	5/5/5
		*Z_r_*	4/4/4	7/7/4	5/4/4	5/7/4	5/7/5	5/7/5	7/4/7
	ML	*a*	5/5/5	5/5/5	5/5/5	5/5/5	5/5/5	7/5/5	7/5/5
		ν	3/5/3	4/5/4	4/5/4	4/7/5	5/7/5	5/7/7	5/7/7
		*t_0_*	4/5/4	5/5/5	5/7/5	5/7/5	5/7/7	5/7/7	7/7/7
		*Z_r_*	4/7/4	5/4/4	7/7/4	7/4/5	5/4/5	7/4/7	7/4/7
	CS	*a*	7/5/5	7/5/5	5/5/5	5/5/5	5/5/5	5/5/3	7/4/7
		ν	3/3/3	3/5/3	4/4/5	5/3/5	4/7/5	7/7/7	7/7/7
		*t_0_*	7/5/5	7/7/7	5/7/5	5/7/5	5/7/7	5/7/7	7/7/7
		*Z_r_*	4/5/4	7/5/4	7/7/7	7/4/7	7/7/7	7/7/7	7/7/7

*The first value is based on the correlation criterion, the second on the bias criterion, and the third on the estimation precision criterion. In the five-parameter model, the intertrial variabilities s_ν_ and s_zr_ are fixed at zero, in the four-parameter model additionally the intertrial variability s_t0_ is fixed at zero and in the three-parameter model also the starting point z_r_ is fixed (z_r_ = 0.5). For conditions with 95% quantiles of parameter estimation precision (weighted against the best possible accuracy) exceeding 25, values are depicted in gray. On the basis of data sets with at least 4% of trials at each threshold*.

One main finding is that in most conditions the seven-parameter model does not provide the most accurate or unbiased estimates, although this is the true model. For ML, the pattern is quite consistent: in most cases, the five-parameter model reveals the best results. For CS, the findings are similar: The five-parameter model shows the best performance. In contrast to the results from ML, the CS procedure more often gets best results from the full seven-parameter model, even for smaller trial numbers. Note, however, that for small trial numbers the performance of CS is generally so poor for all models that results cannot be reasonably interpreted. Therefore, we generally do not recommend using CS for small trial numbers (see also Lerche et al., 2016). For KS more often than for ML and CS, models less complex than the five-parameter model (i.e., the three- or four-parameter models) bring forth the best results. Furthermore, here, more often than for ML and CS, the seven-parameter model performs best. A comparison of the different parameters reveals that for *a* and *t*_0_ the five-parameter model and for *v* and *z*_*r*_ the four-parameter model result in the best recovery.

### Discussion

Study 1 demonstrates that even if the three parameters *a, v*, and *t*_0_ vary from trial to trial (and the starting point is not situated centrally), the seven-parameter model does not always provide the most accurate results.

For data sets with fast contaminants, Lerche et al. (2016) (focusing on the mean precision criterion) showed that a KS based parameter search generally recovers parameters better than ML and CS. Interestingly, in the present analyses, ML and CS show a good performance for data contaminated by fast contaminants, if the five-parameter model is used (see Figure 4). Thus, the inclusion of the intertrial variability of *t*_0_ seems to help to counteract the negative influence of fast contaminants. For KS, on the other hand, a similarly good performance is found for all applied models.

To test the stability of our results, we conducted additional analyses in which the parameter search started with other initial values for the intertrial variabilities. The default initial values of the intertrial variabilities incorporated in *fast-dm* are the following: *s*_ν_ = 0.5; *s*_*zr*_ = 0.3; *s*_*t*0_ = 0.2. In one of the additional estimation series, we set all three intertrial variabilities to zero. In another, we set them to the maximum values used for simulation of data sets (see Table 1). Finally, in a third series of parameter estimation, we set them to half of the maximum values. The main results are very similar for all series of analyses in that the seven-parameter model is mostly outperformed by less complex models.

A caveat of our simulation study is that we made assumptions about the proportion and type of contamination that might not accurately reflect the contamination of real data. We are also not sure about the true range of intertrial variabilities in empirical studies. Another way to analyze the performance of different estimation procedures is provided by a test-retest study.

## Study 2: test-retest study

The main aim of Study 2 was to test whether the conclusions from Study 1 also hold for empirical data. For this purpose, we reanalyzed data from a test-retest study by Lerche and Voss (2016).

### Method

In Study 1 of Lerche and Voss (2016), 105 participants worked at two sessions—separated by 1 week—on an LDT and a Recognition Memory Task (with pictures as stimuli; RMT). As in Study 1 we used *fast-dm-30* and fitted the model using KS, ML, and CS procedures. We also compared the four models differing in complexity as introduced in Study 1. One response (“words” in the LDT and “old pictures” in the RMT) was assigned to the upper threshold, the other response (“non-words” and “new pictures”) to the lower threshold. In each model, we estimated two drift rates (for the different stimulus types). Both drift rates were then combined to an overall measure of speed of information accumulation, termed ν_*total*_ by computing the difference between the drift for words (old pictures) and for non-words (new pictures).

For each of the basic diffusion model parameters (*a*, ν_*total*_*, t*_**0**_, and *z*_*r*_) the Pearson correlation between the two sessions was calculated^5^. To make results more accessible, as in Study 1, the mean over these four coefficients (without *z*_*r*_ in the three-parameter model) was computed using Fisher's Z-transformation (in the remainder of this article termed “mean retest reliability”). Retest correlation coefficients were computed not only for parameters estimated from the actual data (i.e., 200 trials from the RMT, and 400 trials from the LDT), but also for parameters estimated from subsets of data with smaller trial numbers (specifically, for the first 32, 48, 100, and 200 trials of each participant).

Additionally, we wanted to test whether our main findings from Study 1 hold for a different strategy of data simulation. The parameter sets by Lerche et al. (2016) were created using uniform distributions across value ranges typically observed in previous diffusion model studies (only for the drift rates in the two-drift design a multivariate normal distribution was used). Lerche and Voss (2016), on the other hand, based their random parameter sets on multivariate normal distributions defined by the means, standard deviations and correlations of parameter estimates from the data of the LDT and RMT (Table 1). Importantly, as in the simulation study by Lerche et al. (2016), there were substantial intertrial variabilities. Data sets were created using different trial numbers (32—48—100—200—400—1000—5000) and assuming equal parameter sets for both sessions (i.e., no state influences). This allows an estimation of the maximum retest reliability coefficients. Again, in contrast to Lerche and Voss (2016), we estimated parameters using models with different complexity.

### Results

**Figure 5:**
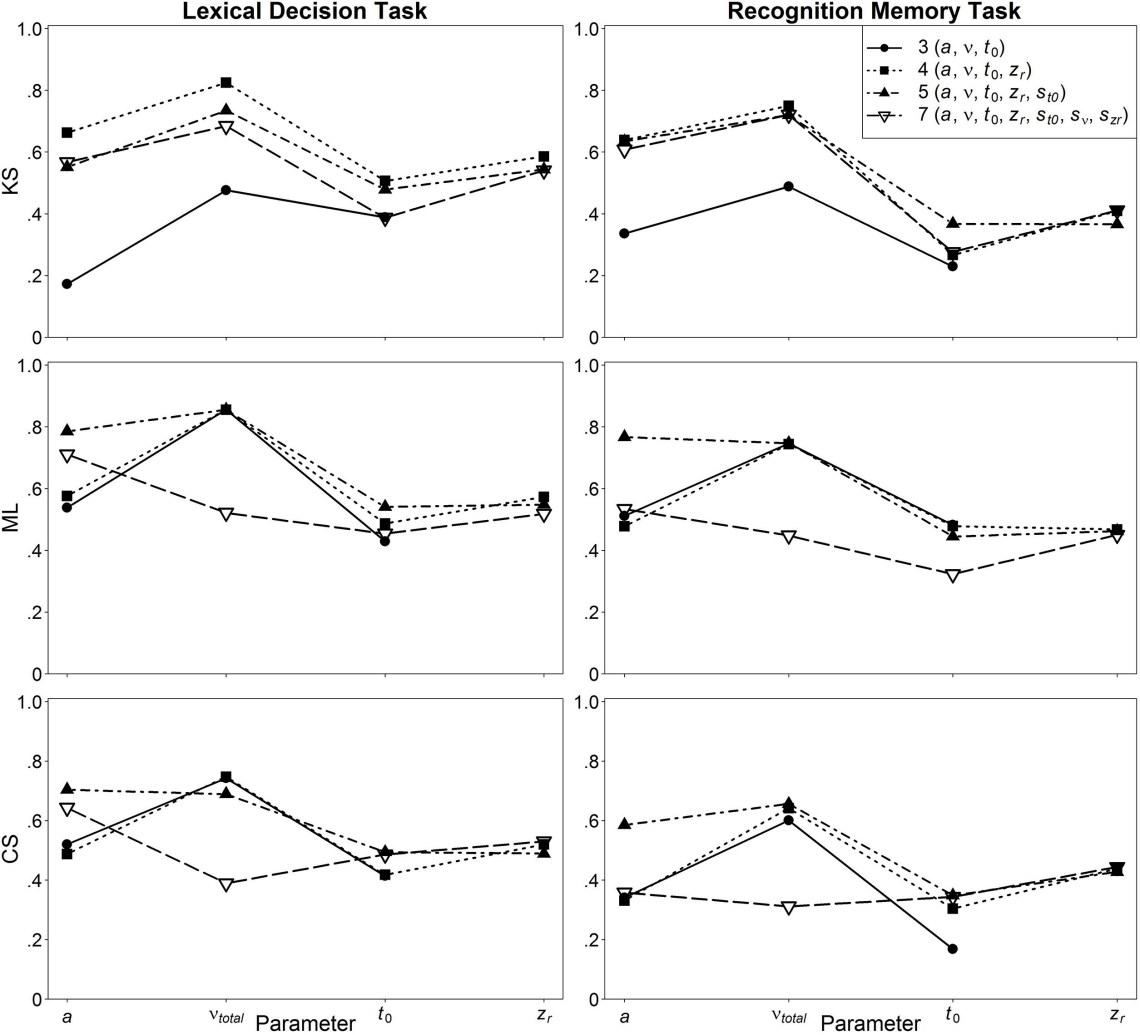
**Retest reliability depending on model complexity and method**.

**Figure 6:**
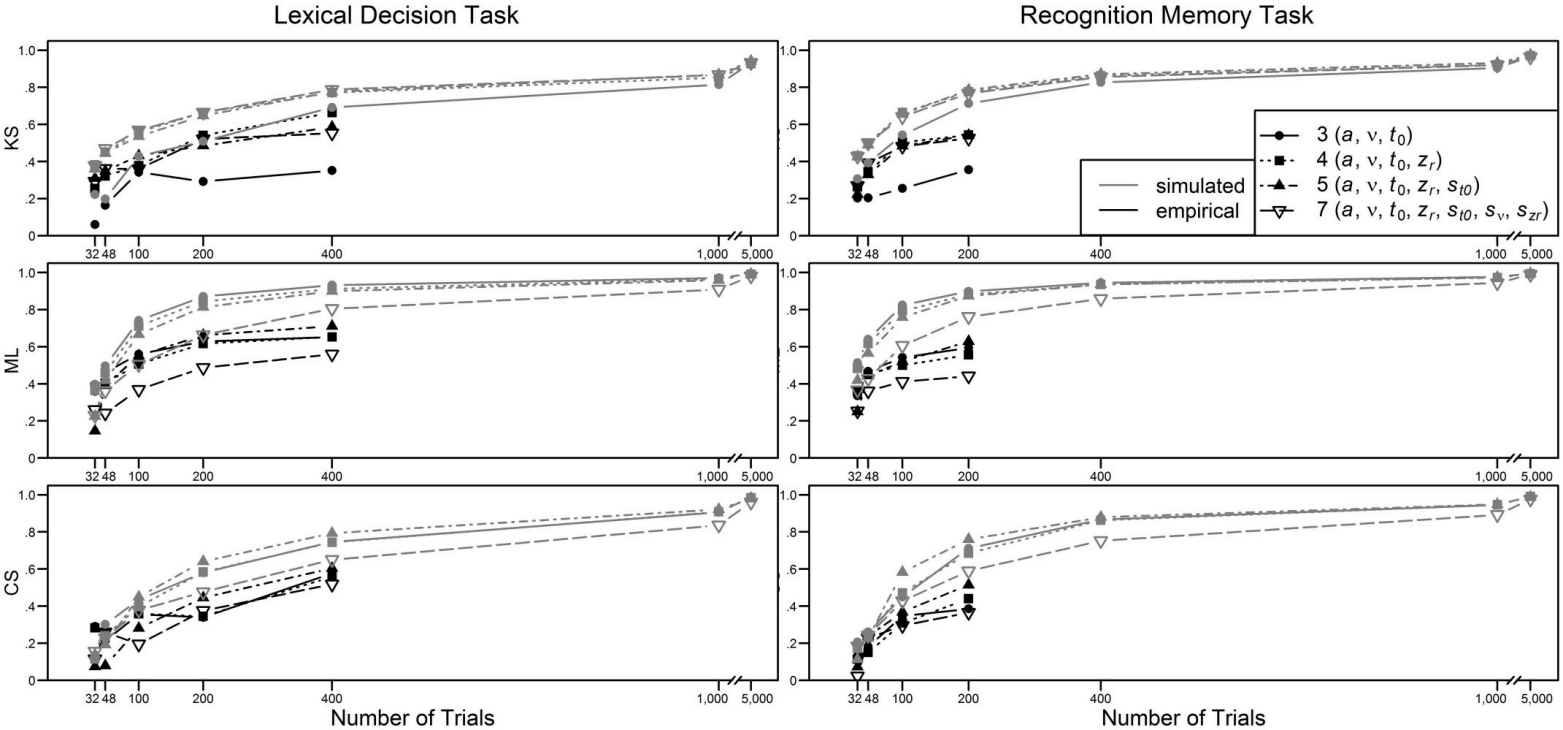
**Mean retest reliability depending on model complexity, method, type of data (empirical vs. simulated), and number of trials**.

In Figure 5, the retest reliabilities are presented for the four main diffusion model parameters for both LDT and RMT as a function of model complexity (estimations are based on the complete data, i.e., 400 and 200 trials for LDT and RMT, respectively). Again, applying the full seven-parameter model does not result in the highest correlations; retest-reliability is higher for less complex models. Whereas, for non-decision time and starting point retest reliabilities for all models are similar, there are larger differences for drift rate and threshold separation. Notable is the poor estimation of drift rates from the seven-parameter model for estimations based on ML or CS. For ML and CS, the five-parameter shows the best performance, whereas for KS, the even more restricted four-parameter model mostly outperforms the other models. Figure 6 shows the influence of the number of trials on retest reliability. Mean reliability coefficients are shown both for the empirical data sets (depicted in black) and the data sets that were simulated on the basis of the parameter ranges observed in the empirical data (depicted in gray). Most importantly, for neither the empirical nor the simulated data does the seven-parameter model show the highest retest correlations. It is noteworthy that for CS and ML, even in the condition with 1000 trials, the seven-parameter model be still worse than the other models^6^.

### Discussion

The main findings from Study 2 are in line with those from Study 1 in that the seven-parameter model does not always show the best performance (here, in terms of the test-retest correlation coefficients). In fact, it is mostly outperformed by less complex models such as the five-parameter model. In the simulation study—which was based on the multivariate distributions of estimated parameters—a similar pattern emerged. This suggests that the main findings do not depend on the particular simulation strategy of Study 1.

Interestingly, using the CS or ML criterion, only at 5000 trials does the seven-parameter model catch up with the more restricted models. Note that sometimes CS has been used for data sets with such high trial numbers. In these studies, the use of a seven-parameter model is justified. Our results, however, suggest that it would be equally effective to use a more restricted model. In addition, it would be more efficient, since the time needed for parameter estimation is prolonged when models with intertrial variabilities are estimated. For smaller trial numbers, on the other hand, the use of the seven-parameter model can lead to worse parameter estimates than the use of more restricted models.

## General discussion

In recent years, an increase in the number of researchers interested in the diffusion model and a higher variability regarding the addressed research topics and experimental designs is evident. For example, while in the past the diffusion model has almost exclusively been used for data sets with very large trial numbers (even >1000; e.g., Ratcliff et al., 2004a; Wagenmakers et al., 2008a; Leite and Ratcliff, 2011), more recently, it has also often been employed for studies with small to moderate trial numbers (e.g., Klauer et al., 2007; Boywitt and Rummel, 2012; Karalunas et al., 2012; Karalunas and Huang-Pollock, 2013; Metin et al., 2013; Pe et al., 2013; Arnold et al., 2015).

Usually, complex models (i.e., with all seven distinct diffusion model parameters and, additionally, parameters varying between several conditions) are used. This has been done even if the number of trials is essentially smaller (e.g., 100 trials, see Metin et al., 2013) than in the studies that originally argued for the inclusion of intertrial variabilities (Ratcliff and Rouder, 1998; Ratcliff and Tuerlinckx, 2002). Especially for small to moderate trial numbers, the choices of model complexity and of optimization criteria for parameter estimation are crucial. Therefore, a systematic comparison of different estimation procedures and a spreading of this knowledge is important in order to support a reasonable use of the diffusion model. With the studies reported here we make a step in this direction.

With two diverse approaches, we analyzed the influence of the model complexity on the accuracy of parameter estimation. We were particularly interested in the influence of the intertrial variabilities (Ratcliff and Rouder, 1998; Ratcliff and Tuerlinckx, 2002) that have proven to be more difficult to estimate than the other diffusion model parameters (e.g., van Ravenzwaaij and Oberauer, 2009). In Study 1, we reanalyzed data sets from a simulation study by Lerche et al. (2016). The data sets were created assuming the presence of intertrial variabilities and a starting point of the diffusion process that was allowed to differ from the center between the thresholds. In Study 2, data from a test-retest study and a further simulation study by Lerche and Voss (2016) were analyzed. While in Study 1 deviations and correlations between the true and the recovered parameter values served as the performance measures, in Study 2 we examined the retest reliability coefficients. In both studies, the parameters were estimated using differently complex models.

Our results for both the simulated and the empirical data sets indicate that the most complex model (the “full” model comprising all seven parameters) is often not the best choice. A five-parameter model (with fixation of *s*_ν_ and *s*_*zr*_ to zero) generally provides accurate estimates, especially when the maximum likelihood (ML) or the chi-square (CS) criterion is applied. For ML and CS, an additional fixation of *s*_*t*0_ is not advisable, since these two criteria are sensitive to the presence of fast contaminants (see also Lerche et al., 2016) and *s*_*t*0_ helps to counteract the negative influence of this type of contamination. Thus, keeping *s*_*t*0_ in the model can help to reach better estimation of the psychologically most interesting parameters (*a*, ν, *t*_0_, and *z*_*r*_). For Kolmogorov-Smirnov (KS)—a criterion that is generally less sensitive to fast contaminants—the even less complex four-parameter model (i.e., the basic diffusion model with all intertrial variabilities fixed at zero) often provides the most accurate results.

Note that our results are in line with recent findings by van Ravenzwaaij et al. (2016). In their study, a model with fixed intertrial variabilities had a higher power to detect differences between conditions than a model including intertrial variabilities. Specifically, results from the EZ approach (Wagenmakers et al., 2007)—which fixes the starting point at the center between the two thresholds and the intertrial variabilities at zero—were compared to the application of a full diffusion model analysis. Even if the data were generated based on a full diffusion model, EZ outperformed the full diffusion model both for detection of drift rate and threshold separation differences. For non-decision time, the efficiency of both procedures was similar.

For future research, it would be interesting to analyze further experimental paradigms using test-retest studies. Besides, one could use different fixation strategies (e.g., instead of fixation at zero, the intertrial variabilities could be fixed at values typically observed in previous studies). To sum up, our results generally speak in favor of the use of less complex models. Thus, if the diffusion model is applied to get accurate estimates of cognitive processes (mapped by *a*, ν, *t*_0_, or *z*_*r*_), a less complex model will often supply more reliable estimates. In particular, it is helpful to fix the intertrial variabilities of starting point and drift rate (*s*_*zr*_ and *s*_ν_) at zero.

## Author contributions

Both VL and AV contributed equally to the conception and interpretation of this work. Data were analyzed by VL and the manuscript was drafted by VL and revised by AV. Both authors approve of the final version and agree on being accountable for this work.

### Conflict of interest statement

The authors declare that the research was conducted in the absence of any commercial or financial relationships that could be construed as a potential conflict of interest. The reviewer VN and the handling Editor declared their shared affiliation, and the handling Editor states that the process nevertheless met the standards of a fair and objective review.

## Footnotes

^1^*Construct-samples* is part of *fast-dm* and offers the possibility of constructing data sets based on a diffusion process.

^2^2In the three-parameter model, the mean was based on *a*, ν, and *t*_0_**. In the two-drift design, first the mean for the criterion performance of the two drift rates was calculated.

^3^“Best possible accuracies” of the main diffusion model parameters: *a* − 0.054; ν − 0.270*; t*_0_ − 0.032*; z*_*r*_ − 0.035. These values are based on an optimal condition of parameter estimation (5000 trials, no contaminants, ML estimation; for more details, please refer to Lerche et al., 2016).

^4^Surprisingly, in some conditions, the estimation precision of KS decreased from 1000 to 5000 trials. This effect is based on a few models with very bad fit that strongly influence the reported 95% quantiles. If medians are examined instead of the 95% quantiles, the estimation precision—as expected—augments from 1000 to 5000 trials, or decreases only marginally. The KS-based search is more prone to get stuck in local minima for larger data sets. Artificial local minima can arise when calculation precision is too low. Exemplarily, we selected the ten data sets that showed the worst performance in the condition with 5000 trials in the one-drift model with no contaminants. We then reestimated parameters for these data sets with the seven-parameter model with increased precision of calculation (the fast-dm precision criterion was increased from 3 to 4). This improved parameter estimation notably for the condition with 5000 trials. More specifically, the mean across these ten data sets dropped to less than half, whereas there was less improvement for the condition with 1000 trials. Accordingly, for higher trial numbers, we recommend using higher precision settings in fast-dm.

^5^Prior to the correlational analyses, we identified bivariate outliers with the Mahalanobis distance (*D*^2^) and excluded participants with extremely high values (*p* < 0.001) from the respective analysis (resulting in at most four excluded participants).

^6^Note that we also analyzed the Associative Priming Task presented in Lerche and Voss (2016; Study 2) using models with different complexity. We found very similar results in that the seven-parameter model did not show the highest retest reliabilities.
